# Generation of an ultra-short electrical pulse with width shorter than the excitation laser

**DOI:** 10.1038/srep27577

**Published:** 2016-06-08

**Authors:** Wei Shi, Shaoqiang Wang, Cheng Ma, Ming Xu

**Affiliations:** 1 Applied Physics Department, Xi’an University of Technology, Xi’an, 710048, P. R. China

## Abstract

We demonstrate experimentally a rare phenomenon that the width of an electrical response is shorter than that of the excitation laser. In this work, generation of an ultrashort electrical pulse is by a semi-insulating GaAs photoconductive semiconductor switch (PCSS) and the generated electrical pulse width is shorter than that of the excitation laser from diode laser. When the pulse width and energy of the excitation laser are fixed at 25.7 ns and 1.6 μJ respectively, the width of the generated electrical pulse width by 3-mm-gap GaAs PCSS at the bias voltage of 9 kV is only 7.3 ns. The model of photon-activated charge domain (PACD) is used to explain the peculiar phenomenon in our experiment. The ultrashort electrical pulse width is mainly relevant to the time interval of PACD from occurrence to disappearance in the mode. The shorter the time interval is, the narrower the electrical pulse width will become. In more general terms, our result suggests that in nonlinear regime a response signal can have a much short width than the excitation pulses. The result clearly indicates that generating ultrashort electrical pulses can be achieved without the need of ultrashort lasers.

Gallium arsenide photoconductive semiconductor switch is an excellent optically-activated pulsed power device^1^,^2^,^3^,^4^. It has many advantages over the conventional electrically-triggered device, such as simple structure, fast response, low jitter and high repetition rate^1^,^5^. It has the potential to significantly increase the switching frequencies for all power levels and is capable of generating high-power ultrashort pulses^1^,^6^. It is widely known that GaAs PCSS has two operating modes, the linear mode and the high-gain mode (also called “lock-on” mode)^7^,^8^. In the linear mode, an ultrashort electric pulse is generated by a desktop laser, but transmission efficiency of the bias voltage is low, and the bulky desktop laser is not suitable for integration^6^,^9^. Under certain conditions of optical and electrical thresholds, GaAs PCSS triggered by a compact laser diode (LD) operates in the high-gain mode^10^. In this mode, when illuminated by a laser, the switch will turn on and stay on (lock-on) until most of the energy is discharged^7^,^8^. And the voltage across the switch will drop to the constant but nonzero value, the lock-on voltage, which is independent of the initial charging voltage^7^,^11^,^12^. The self-sustained ‘on’ state is called lock-on phenomenon. When the sustained “lock-on” phenomenon occurs, its associated current filaments often lead to reducing operation frequency of the switch and restricting devices’ lifetime^13^,^14^. In order to improve the performance of GaAs PCSS, a number of methods have been used to avoid the lock-on state, including the use of a series transmission line circuit^15^, a parallel transmission line circuit^15^, a series inductor in circuit^15^, a series spark gap^16^, a double-layer GaAs PCSS^17^ and so on. However, there are some disadvantages about complex circuits such as unstable operation and wider pulse width. In ref. 18, it is experimentally demonstrated that the high-gain mode can be quenched due to the mechanism of photon-activated charge domain (PACD)^18^,^19^. In this mechanism, the high gain mode will be quenched when the electrical field across a PCSS is lower than the minimum electrical field required to maintain the high-gain mode^18^. Thus, in order to quench the high-gain mode, a small storage capacitor is used to control bias electrical field in our experiment. We found a rare phenomenon that the width of output electrical pulse generated by GaAs PCSS is shorter than the width of excitation laser from LD. An electrical pulse with the full width at half maximum (FWHM) of 7.3 ns is generated when GaAs PCSS is illuminated by LD with the FWHM of 25.4 ns. The reasons why an electrical pulse is shorter than the excitation pulse is discussed using the quenched high-gain mode and the PACD.

## Methods

### GaAs PCSS

**Figure 1:**
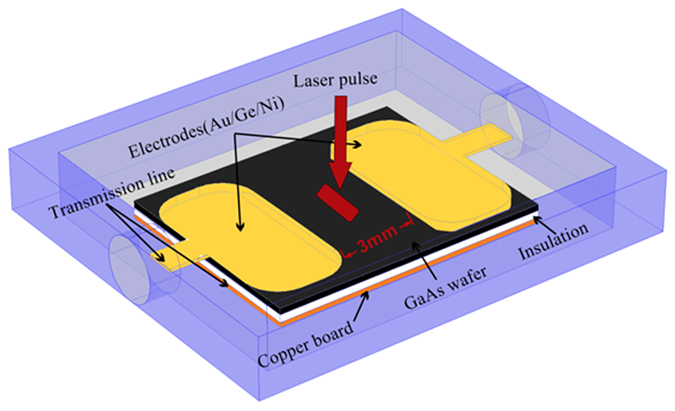
Schematic diagram of the lateral GaAs PCSS.

In our experiment, the semi-insulating (SI) GaAs is used to design PCSS, with a resistivity of 5 × 10^7 ^Ω·cm in total darkness, and the electron mobility is larger than 5500 cm^2^/(V·s). The size of the GaAs PCSS is 8.0 mm (width) × 10.0 mm (length) × 0.6 mm (thickness), and the distance between the two electrodes is 3 mm. The Au/Ge/Ni alloy electrodes with ohmic contact are deposited on the surface of the photoconductive material by the electron beam evaporation technique. The size of each electrode is 6 mm × 3 mm, and a fillet with radius of 1.1 mm is designed to improve electric field distribution. A 900 nm Si_3_N_4_ film is deposited on the surface of substrate as a passivation layer. The transparent solid medium organic silicon gel, the insulation strength of which exceeds 289 kV/cm, is used for insulation protection. The GaAs PCSS is installed on the substrate of a copper board with transmission line that is connected to external circuits by two coaxial output connectors. Finally, the lateral solid-state GaAs PCSS is placed on the metal box to provide physical protection. The schematic device design is shown in Fig. 1.

### The measurement circuit

**Figure 2:**
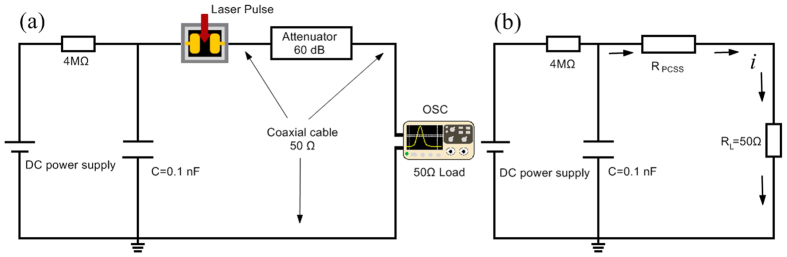
(**a**) Schematic diagram of measurement circuit for the PCSS. (**b**) Lumped-parameter circuit model of measurement circuit for the PCSS.

The measurement circuit is shown in Fig. 2a. And 0.1 nF ceramic capacitor is charged by the high-voltage DC source through a 4 MΩ resistor, providing the GaAs PCSS discharge circuit with sufficient power. The output electrical pulse is attenuated by an attenuator (60 dB), and then measured by a digital storage oscilloscope (LeCroy WAVERUNNER 64Xi) with the bandwidth of 600 MHz.

### Laser diode

**Figure 3:**
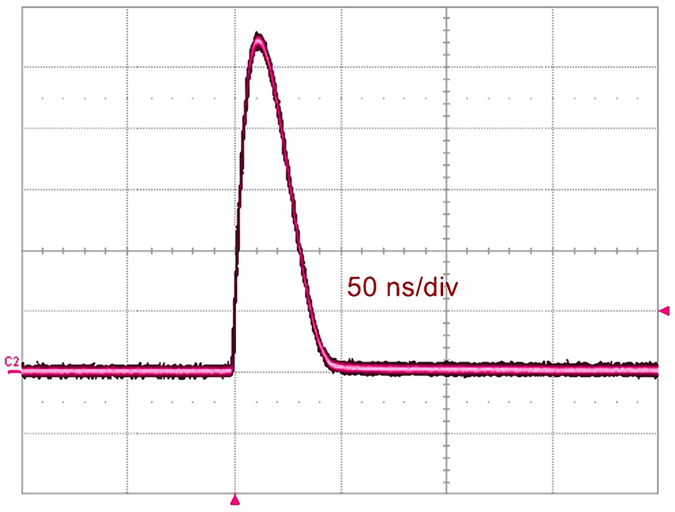
One hundred times superposed waveforms of LD (SPL PL90-3). The average width time is 25.7 ns. The average rise time is 7.1 ns.

An LD (SPL PL90-3 nanosecond pulse laser diode) is used in our experiment. The output laser is characterized by 905 nm wavelength, 25.7 ns pulsewidth, rise time of 7.1 ns, and single pulse energy of 1.6 μJ, as shown in the Fig. 3. Considering the influence of beam divergence angle and the distance between the LD head and the surface of the switch, the shape of trigger spot is considered to be a rectangular region and the area is 8.31 mm^2^ in the middle of the two electrodes, which is displayed in Fig. 1.

## Results and Discussions

**Figure 4:**
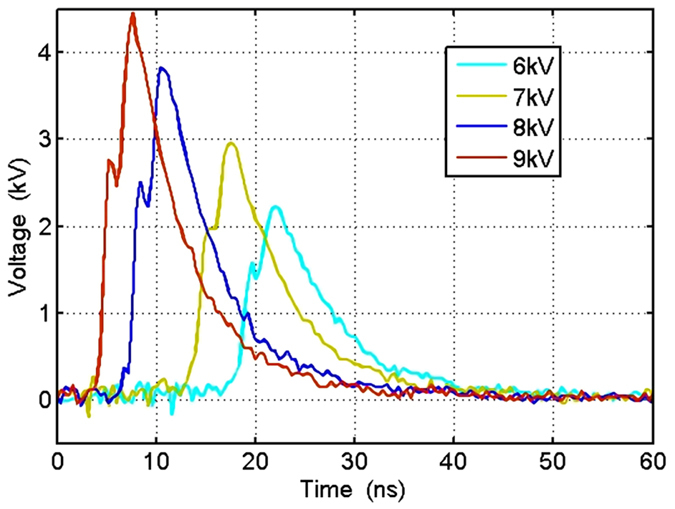
The output waveforms of the 3-mm-gap GaAs PCSS at the bias voltage from 6 kV to 9 kV with a step of 1 kV. When t = 0 ns, the GaAs PCSS is illuminated.

We demonstrated an electrical pulse of width shorter than that of incident laser can be generated by the GaAs PCSS in the quenched high-gain mode^18^. The new mode has both advantages of the high-gain mode and linear mode^20^. As shown in Fig. 4, when the bias voltage is 9 kV (the bias electrical field is 30 kV/cm), the rise time of output electrical waveform is 3 ns, the FWHM is 7.3 ns and the amplitude is 4.46 kV. The FWHM of output electrical waveform is about 1/3 of that of incident laser, and it is the same with the rise time. The relationship between bias voltage and the output electrical waveform is shown in Fig. 4. Apparently, the delay time decreases as the bias voltage increases when the GaAs PCSS is illuminated at t = 0 ns, and the rise time is 4.67 ns, 4 ns, 3.56 ns and 3 ns from 6 kV to 9 kV respectively, all are shorter than the optical rise time of 7.1 ns. Although the phenomena described above belong to the characteristics of high-gain mode^12^,^21^,^22^, the electrical pulse width is shorter than the optical pulse width without lock-on phenomenon at the bias voltage from 6 kV to 9 kV. Hence, this is a new stabilized mode which is called the quenched high-gain mode^18^.

In the later section, the reasons for the shorter electrical pulse are discussed based on the principle of PACD. The calculation results show that the lower bound of electrical field for maintaining the GaAs PCSS in high-gain mode is about 7.5 kV/cm. Compared with the incident laser, the electrical width is closely related to the time interval of PACD from occurrence to disappearance in the quenched high-gain mode, and the shorter the time interval is, the narrower the electrical width will become. In summary, the reasons why a shorter electrical pulse is generated is that the PACD is formed and quenched quickly.

The lumped-parameter mode of the experimental circuit is shown in Fig. 2b. There is a 4 MΩ resistor to limit the charging currents for protecting power source. When the GaAs PCSS is illuminated, the charging time of the capacitor (0.1 nF) is much longer than the discharging time in this circuit. So we can ignore the charging in our calculation. In the high-gain mode, the electrical field across the switch will drop to a relatively constant, the lock-on field, which is independent of the optical trigger energy, the switch length, and the initial voltage across the switch^7^,^8^. However, if the energy in the capacitance can not maintain the lock-on field in the sustaining phase, the current will decrease steeply in the circle^23^. Thus, the current in the circle and the electrical field across the switch should be calculated in our equations. In our calculation, the discrete-time voltages of 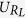 on the load *R*_*L*_ are acquired in the storage oscilloscope (see Supplementary Table S1), as shown in Fig. 4. △t represents the time interval of discrete voltage, and ‘n’ represents the number of discrete voltage. These constraint equations are formulated based on the principle of charge conservation,


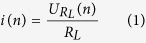



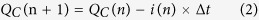



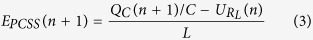



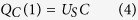


where *Q*_*C*_, *E*_*PCSS*_, *i*, *U*_*S*_ and *L* are the quantity of electric charge in the capacitance C, the transient average electrical field across the PCSS, the transient current in circuit, the initial charging voltage and the distance between the two electrodes, respectively. In these equations, the current waveforms are calculated by Eq. (1), the residual charge quantity as a function of time is calculated by Eq. (2), the transient electrical field across the switch is calculated by Eq. (3), and Eq. (4) is an initial condition.

**Figure 5:**
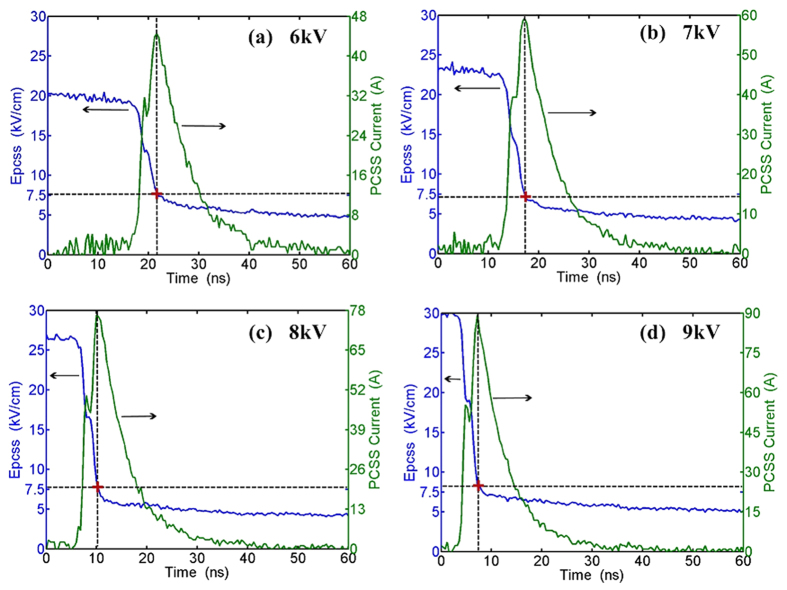
The average electrical filed across the GaAs PCSS is shown in the left ordinate, the current waveform is shown in the right ordinate, when the bias voltage is at 6 kV,7 kV, 8 kV, 9 kV.

Every discrete voltage, in turn, is plugged into Eq. (1) to Eq. (4). Finally, the transient current and the average electrical field across GaAs PCSS is calculated at the bias voltage from 6 kV to 9 kV, as shown in Fig. 5. The vertical and lateral auxiliary dashed lines display the moment and the lock-on electrical field when the switch exits from the high-gain mode. The crossover points of dashed lines present the high-gain quenched threshold (as marked ‘+’ in figures).

The calculation results show the quenched threshold is about 7.5 kV/cm in Fig. 5(a–d), which represents the minimum electrical field for maintaining the high-gain mode in the GaAs PCSS^20^. Once the average electrical field decreases to the critical value, the switch will quickly turn off. Meanwhile, the quenched high-gain threshold equals the threshold of the Gunn Effect. The Gunn threshold field of different materials is not the same^24^, and neither is the quenched high-gain threshold field. Thus, it is significant to investigate the process of forming the ultrashort pulses for improving performance of switch. Illuminated by laser, the PACD is formed in the switch after a certain delay time, and the switch will operate in high-gain mode. If the electrical field across the switch is sustainable higher than the high-gain quenched threshold, the lock-on phenomenon will occur and result in low repetition rate of GaAs PCSS. In order to improve the repetition rate, we utilize a small capacitance to rapidly decrease the electrical field across the switch to the critical value (7.5 kV/cm). In this case, the switch exits from high-gain mode and works again in linear mode. Additionally, the moment when the switch exits from the high-gain mode is very sensitive to the bias voltage. And it decreases from more than 20 ns to less than 10 ns when the bias increases from 6 kV to 9 kV. The main reason for the phenomena is that the delay time is very sensitive to the bias. And the delay time is defined as the time interval between the beginning of optical illumination and the onset of switching in photocurrent in GaAs PCSS^21^. With the increasing bias voltage, the delay time is changed from 17 ns to 4 ns in our experiment, as shown in Fig. 5.

**Figure 6:**
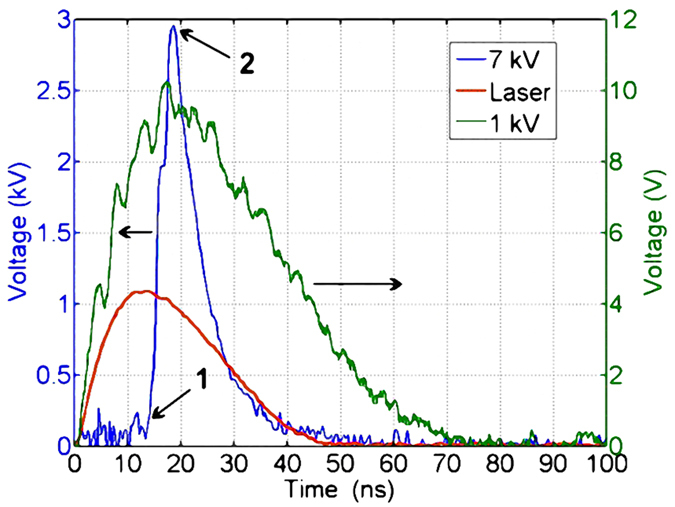
The output waveform of GaAs PCSS is shown in the left ordinate at the bias voltage of 7 kV. The output waveform of GaAs PCSS is shown in the right ordinate at the bias voltage of 1 kV. The red curve represents the incident laser.

Figure 6 clearly reveals that the output electrical width is shorter than the laser width at the bias voltage of 7 kV. Because the linear voltage is much weaker than the nonlinear voltage, we use a fixed X-axis and a dual Y-axis coordinate system where X is time and Y is amplitude. The duration time of laser means the illuminating time. Output electrical pulse under the applied voltage of 1 kV is only used to mark the time at which the optical trigger reaches the PCSS. Because the applied voltage of 1 kV is lower than the voltage threshold of 4.8 kV (the threshold voltage of high-gain mode in our experiment), the GaAs PCSS operates in linear mode without delay time. Since the phenomena are similar at different bias voltage in our experiment, only the bias voltage of 7 kV is used to illustrate our experimental phenomenon.

In Fig. 6, after the delay time of 13.4 ns, the waveform begins to rise steeply at the applied voltage of 7 kV when the PACD is formed in the switch. The symbol ‘1’ marks the onset of this steep rise. The symbol ‘2’ marks the moment when the PACD is quenched (see Fig. 5b). At ‘2’ the average field across the switch is lower than the high-gain quenched threshold. The left region of ‘1’represents the delay time in the high-gain mode, and the right region of ‘2’has the meaning of recovery time in linear mode. The region between ‘1’ and ‘2’ shows evolution of PACD from occurrence to disappearance, which also reflects the process of the rising edge of output pulse. In other words, when the amplitude of output pulse is rising, the PACD is quenched, and meanwhile the switch enters again into linear mode. However, the fall time is dependent on the nonequilibrium carrier lifetime in the linear mode, and the nonequilibrium carrier recombination is extremely fast. Thus, the switch quickly turns off due to carrier recombination^7^,^25^. Finally, an ultrashort electrical pulse is generated without the lock-on phenomenon. However, if the PACD is quenched in the lock-on state, the width of electrical pulse may be broadened, or even a square-wave pulse is generated^20^. Thus, the electrical pulse width is mainly relevant to the time interval from ‘1’ to ‘2’ in the high-gain quenched mode, and if the time interval becomes shorter, the electrical width will become narrower.

In conclusion, because the PACD is formed and quenched quickly, an electrical pulse whose width is shorter than that of the incident laser is generated by GaAs PCSS. More generally our result suggests that it is possible to generate a response signal whose width is shorter than that of the excitation signal. This result paves the way for developing techniques of ultrashort pulse generation without the need of ultrashort excitation lasers, making the ultrashort electrical pulse more accessible.

## Additional Information

**How to cite this article**: Shi, W. *et al.* Generation of an ultra-short electrical pulse with width shorter than the excitation laser. *Sci. Rep.* **6**, 27577; doi: 10.1038/srep27577 (2016).

## Supplementary Material

Supplementary Information
