# Supplementary material for: Generation of an ultra-short electrical pulse with width shorter than the excitation laser
Source: Sci Rep. 2016 Jun 8;6:27577. doi: 10.1038/srep27577 (PMC4897789; doi:10.1038/srep27577)
Supplement: Supplementary Information [file srep27577-s1.pdf]

Generation of an ultra-short electrical pulse with width shorter than the excitation laser

Wei Shi*, Shaoqiang Wang, Cheng Ma and Ming Xu

Applied Physics Department, Xi'an University of Technology, Xi'an, 710048, P. R. China

*Corresponding Author:

Wei Shi

Applied Physics Department

Xi'an University of Technology

Xi'an, 710048

P. R. China

E-mail: swshi@mail.xaut.edu.cn

Supplementary data

Supplementary Table S1: The four sets of data points about the output waveform, when the bias voltage is at 6kV, 7kV, 8kV and 9kV.

6kV		7kV		8kV		9kV	
Time	Ampl	Time	Ampl	Time	Ampl	Time	Ampl
-4.02E-08	0.048075	-4.01E-08	0.0160762	-4.00E-08	-0.0159226	-4.03E-08	0.0160762
-3.98E-08	-0.0159226	-3.97E-08	0.0160762	-3.96E-08	0.048075	-3.99E-08	0.048075
-3.94E-08	-0.0159226	-3.93E-08	-0.0159226	-3.92E-08	-0.0479214	-3.95E-08	0.0160762
-3.90E-08	0.0160762	-3.89E-08	0.0160762	-3.88E-08	0.0160762	-3.91E-08	0.0160762
-3.86E-08	-0.0159226	-3.85E-08	0.048075	-3.84E-08	0.0160762	-3.87E-08	0.0160762
-3.82E-08	-0.0159226	-3.81E-08	-0.0479214	-3.80E-08	0.048075	-3.83E-08	-0.0159226
-3.78E-08	0.0160762	-3.77E-08	-0.0159226	-3.76E-08	0.0160762	-3.79E-08	-0.0159226
-3.74E-08	-0.0479214	-3.73E-08	0.0160762	-3.72E-08	-0.0159226	-3.75E-08	0.048075
-3.70E-08	-0.0159226	-3.69E-08	-0.0159226	-3.68E-08	0.0160762	-3.71E-08	0.048075
-3.66E-08	-0.0159226	-3.65E-08	-0.0159226	-3.64E-08	-0.0159226	-3.67E-08	0.048075
-3.62E-08	-0.0159226	-3.61E-08	0.0160762	-3.60E-08	0.0160762	-3.63E-08	0.048075
-3.58E-08	-0.0159226	-3.57E-08	-0.0159226	-3.56E-08	-0.0159226	-3.59E-08	0.048075
-3.54E-08	0.0160762	-3.53E-08	-0.0159226	-3.52E-08	0.0160762	-3.55E-08	0.0160762
-3.50E-08	0.0160762	-3.49E-08	0.0800737	-3.48E-08	0.048075	-3.51E-08	0.048075
-3.46E-08	-0.0479214	-3.45E-08	0.0160762	-3.44E-08	-0.0159226	-3.47E-08	0.0160762
-3.42E-08	0.048075	-3.41E-08	-0.0159226	-3.40E-08	-0.0159226	-3.43E-08	-0.0159226
-3.38E-08	-0.0479214	-3.37E-08	0.0160762	-3.36E-08	0.0160762	-3.39E-08	-0.0479214
-3.34E-08	-0.0799201	-3.33E-08	-0.0479214	-3.32E-08	0.0160762	-3.35E-08	-0.0479214
-3.30E-08	0.0160762	-3.29E-08	-0.0159226	-3.28E-08	-0.0159226	-3.31E-08	0.048075
-3.26E-08	0.0160762	-3.25E-08	-0.0479214	-3.24E-08	0.048075	-3.27E-08	-0.0159226
-3.22E-08	-0.0159226	-3.21E-08	-0.0479214	-3.20E-08	-0.0799201	-3.23E-08	-0.0159226
-3.18E-08	0.0160762	-3.17E-08	-0.0159226	-3.16E-08	-0.0479214	-3.19E-08	0.048075
-3.14E-08	0.048075	-3.13E-08	-0.0479214	-3.12E-08	-0.0159226	-3.15E-08	-0.0159226
-3.10E-08	-0.0479214	-3.09E-08	-0.0479214	-3.08E-08	0.048075	-3.11E-08	0.0160762
-3.06E-08	-0.0159226	-3.05E-08	0.0160762	-3.04E-08	0.0160762	-3.07E-08	-0.0159226
-3.02E-08	0.0160762	-3.01E-08	-0.0159226	-3.00E-08	-0.0159226	-3.03E-08	-0.0799201
-2.98E-08	-0.0159226	-2.97E-08	-0.0159226	-2.96E-08	-0.0159226	-2.99E-08	-0.0479214
-2.94E-08	-0.0159226	-2.93E-08	0.048075	-2.92E-08	-0.0479214	-2.95E-08	-0.0159226
-2.90E-08	0.048075	-2.89E-08	-0.0159226	-2.88E-08	-0.0159226	-2.91E-08	-0.0159226
-2.86E-08	-0.0479214	-2.85E-08	-0.0159226	-2.84E-08	-0.0159226	-2.87E-08	-0.0159226
-2.82E-08	-0.0159226	-2.81E-08	-0.0159226	-2.80E-08	-0.0799201	-2.83E-08	0.0160762
-2.78E-08	-0.0159226	-2.77E-08	0.0160762	-2.76E-08	-0.0479214	-2.79E-08	0.0160762
-2.74E-08	-0.0799201	-2.73E-08	-0.0159226	-2.72E-08	0.048075	-2.75E-08	0.048075
-2.70E-08	0.0160762	-2.69E-08	0.0160762	-2.68E-08	-0.0159226	-2.71E-08	0.048075
-2.66E-08	-0.0159226	-2.65E-08	-0.0159226	-2.64E-08	-0.0159226	-2.67E-08	0.0160762
-2.62E-08	0.0160762	-2.61E-08	-0.0479214	-2.60E-08	0.0160762	-2.63E-08	-0.0159226

-2.58E-08	0.0160762	-2.57E-08	-0.0159226	-2.56E-08	-0.0159226	-2.59E-08	0.0160762
-2.54E-08	-0.0159226	-2.53E-08	0.048075	-2.52E-08	0.0160762	-2.55E-08	-0.0159226
-2.50E-08	-0.0479214	-2.49E-08	0.048075	-2.48E-08	-0.0159226	-2.51E-08	-0.0159226
-2.46E-08	-0.0159226	-2.45E-08	0.0160762	-2.44E-08	-0.0479214	-2.47E-08	0.0160762
-2.42E-08	0.0160762	-2.41E-08	-0.0159226	-2.40E-08	0.0160762	-2.43E-08	-0.0159226
-2.38E-08	0.0160762	-2.37E-08	-0.0479214	-2.36E-08	0.048075	-2.39E-08	-0.0479214
-2.34E-08	-0.0159226	-2.33E-08	0.0160762	-2.32E-08	-0.0159226	-2.35E-08	-0.0159226
-2.30E-08	-0.0159226	-2.29E-08	-0.0479214	-2.28E-08	0.0160762	-2.31E-08	0.0160762
-2.26E-08	-0.0479214	-2.25E-08	-0.0799201	-2.24E-08	-0.0159226	-2.27E-08	-0.0159226
-2.22E-08	0.0160762	-2.21E-08	0.0160762	-2.20E-08	-0.0479214	-2.23E-08	0.0160762
-2.18E-08	0.0160762	-2.17E-08	-0.0479214	-2.16E-08	-0.0479214	-2.19E-08	-0.0479214
-2.14E-08	0.0160762	-2.13E-08	-0.0479214	-2.12E-08	-0.0159226	-2.15E-08	-0.0159226
-2.10E-08	0.0160762	-2.09E-08	0.0160762	-2.08E-08	-0.0479214	-2.11E-08	0.048075
-2.06E-08	-0.0159226	-2.05E-08	0.0160762	-2.04E-08	-0.0479214	-2.07E-08	-0.0159226
-2.02E-08	-0.0159226	-2.01E-08	-0.0479214	-2.00E-08	-0.0159226	-2.03E-08	-0.0159226
-1.98E-08	0.0160762	-1.97E-08	0.0160762	-1.96E-08	-0.0159226	-1.99E-08	-0.0159226
-1.94E-08	0.048075	-1.93E-08	-0.0159226	-1.92E-08	0.0160762	-1.95E-08	0.0160762
-1.90E-08	-0.0159226	-1.89E-08	-0.0159226	-1.88E-08	0.0800737	-1.91E-08	-0.0159226
-1.86E-08	0.0160762	-1.85E-08	-0.0479214	-1.84E-08	0.0160762	-1.87E-08	-0.0159226
-1.82E-08	-0.0159226	-1.81E-08	-0.111919	-1.80E-08	0.0160762	-1.83E-08	-0.0159226
-1.78E-08	-0.0479214	-1.77E-08	-0.111919	-1.76E-08	-0.0479214	-1.79E-08	-0.0479214
-1.74E-08	0.0160762	-1.73E-08	0.048075	-1.72E-08	-0.0159226	-1.75E-08	-0.0159226
-1.70E-08	0.0800737	-1.69E-08	-0.0159226	-1.68E-08	0.0160762	-1.71E-08	-0.0159226
-1.66E-08	-0.0479214	-1.65E-08	-0.0479214	-1.64E-08	0.0160762	-1.67E-08	-0.0159226
-1.62E-08	0.0160762	-1.61E-08	0.048075	-1.60E-08	-0.0159226	-1.63E-08	0.0160762
-1.58E-08	-0.0479214	-1.57E-08	0.0160762	-1.56E-08	-0.0159226	-1.59E-08	-0.0799201
-1.54E-08	-0.0159226	-1.53E-08	-0.0799201	-1.52E-08	-0.0479214	-1.55E-08	-0.0479214
-1.50E-08	0.0160762	-1.49E-08	-0.0479214	-1.48E-08	-0.0479214	-1.51E-08	0.048075
-1.46E-08	0.0160762	-1.45E-08	-0.0159226	-1.44E-08	-0.0159226	-1.47E-08	-0.0159226
-1.42E-08	-0.0159226	-1.41E-08	-0.0159226	-1.40E-08	-0.0479214	-1.43E-08	-0.0159226
-1.38E-08	0.048075	-1.37E-08	-0.0479214	-1.36E-08	-0.0479214	-1.39E-08	-0.0159226
-1.34E-08	-0.0479214	-1.33E-08	0.0160762	-1.32E-08	0.0160762	-1.35E-08	-0.0479214
-1.30E-08	-0.0159226	-1.29E-08	-0.0159226	-1.28E-08	-0.0159226	-1.31E-08	0.112072
-1.26E-08	-0.0159226	-1.25E-08	0.048075	-1.24E-08	-0.0159226	-1.27E-08	0.0160762
-1.22E-08	0.0160762	-1.21E-08	-0.0159226	-1.20E-08	0.048075	-1.23E-08	-0.143918
-1.18E-08	-0.0159226	-1.17E-08	-0.0159226	-1.16E-08	0.0160762	-1.19E-08	0.048075
-1.14E-08	0.0160762	-1.13E-08	-0.0159226	-1.12E-08	-0.0479214	-1.15E-08	-0.111919
-1.10E-08	0.048075	-1.09E-08	-0.0479214	-1.08E-08	0.0160762	-1.11E-08	0.0800737
-1.06E-08	-0.0159226	-1.05E-08	-0.0479214	-1.04E-08	-0.0799201	-1.07E-08	-0.143918
-1.02E-08	-0.0799201	-1.01E-08	-0.0159226	-1.00E-08	-0.0159226	-1.03E-08	0.048075
-9.78E-09	-0.0479214	-9.66E-09	-0.0479214	-9.64E-09	-0.0799201	-9.93E-09	0.144071
-9.38E-09	-0.0479214	-9.26E-09	-0.0479214	-9.24E-09	0.112072	-9.53E-09	-0.143918
-8.98E-09	-0.0159226	-8.86E-09	0.0160762	-8.84E-09	0.0160762	-9.13E-09	0.144071

-8.58E-09	-0.0159226	-8.46E-09	0.048075	-8.44E-09	0.112072	-8.73E-09	-0.143918
-8.18E-09	-0.0799201	-8.06E-09	0.048075	-8.04E-09	0.112072	-8.33E-09	0.0160762
-7.78E-09	-0.0159226	-7.66E-09	-0.0159226	-7.64E-09	0.0160762	-7.93E-09	-0.111919
-7.38E-09	-0.0159226	-7.26E-09	-0.0159226	-7.24E-09	0.0160762	-7.53E-09	-0.239914
-6.98E-09	-0.0479214	-6.86E-09	-0.0159226	-6.84E-09	-0.0479214	-7.13E-09	0.17607
-6.58E-09	0.0160762	-6.46E-09	0.0160762	-6.44E-09	-0.271913	-6.73E-09	-0.0799201
-6.18E-09	0.0160762	-6.06E-09	-0.0159226	-6.04E-09	-0.0479214	-6.33E-09	0.144071
-5.78E-09	-0.0159226	-5.66E-09	-0.0159226	-5.64E-09	-0.143918	-5.93E-09	-0.207915
-5.38E-09	0.0160762	-5.26E-09	0.0160762	-5.24E-09	0.048075	-5.53E-09	-0.143918
-4.98E-09	-0.0159226	-4.86E-09	0.048075	-4.84E-09	-0.175916	-5.13E-09	0.048075
-4.58E-09	-0.0159226	-4.46E-09	-0.0159226	-4.44E-09	0.0800737	-4.73E-09	-0.0799201
-4.18E-09	0.0160762	-4.06E-09	-0.0159226	-4.04E-09	0.048075	-4.33E-09	0.112072
-3.78E-09	0.0160762	-3.66E-09	0.112072	-3.64E-09	0.048075	-3.93E-09	-0.0479214
-3.38E-09	-0.0159226	-3.26E-09	0.0160762	-3.24E-09	0.0800737	-3.53E-09	0.0160762
-2.98E-09	-0.0479214	-2.86E-09	0.048075	-2.84E-09	-0.0799201	-3.13E-09	-0.0159226
-2.58E-09	0.0160762	-2.46E-09	0.048075	-2.44E-09	0.048075	-2.73E-09	0.0160762
-2.18E-09	-0.0159226	-2.06E-09	-0.0479214	-2.04E-09	0.0160762	-2.33E-09	0.144071
-1.78E-09	-0.0159226	-1.66E-09	0.0160762	-1.64E-09	0.048075	-1.93E-09	0.0160762
-1.38E-09	0.0160762	-1.26E-09	-0.0159226	-1.24E-09	0.0800737	-1.53E-09	-0.143918
-9.79E-10	0.048075	-8.60E-10	0.048075	-8.37E-10	-0.111919	-1.13E-09	-0.0159226
-5.79E-10	-0.0479214	-4.60E-10	0.048075	-4.37E-10	0.0800737	-7.30E-10	-0.0159226
-1.79E-10	-0.0159226	-5.99E-11	0.112072	-3.69E-11	-0.0479214	-3.30E-10	0.112072
2.21E-10	-0.0159226	3.40E-10	0.048075	3.63E-10	0.144071	7.03E-11	0.0800737
6.21E-10	-0.0159226	7.40E-10	0.048075	7.63E-10	0.112072	4.70E-10	0.048075
1.02E-09	0.0160762	1.14E-09	0.112072	1.16E-09	0.112072	8.70E-10	0.17607
1.42E-09	-0.0159226	1.54E-09	-0.0159226	1.56E-09	0.112072	1.27E-09	-0.0479214
1.82E-09	0.048075	1.94E-09	0.048075	1.96E-09	-0.0799201	1.67E-09	-0.0159226
2.22E-09	-0.0159226	2.34E-09	0.112072	2.36E-09	0.0160762	2.07E-09	0.048075
2.62E-09	-0.0159226	2.74E-09	-0.207915	2.76E-09	0.112072	2.47E-09	0.112072
3.02E-09	0.048075	3.14E-09	0.272066	3.16E-09	0.112072	2.87E-09	0.112072
3.42E-09	0.0800737	3.54E-09	0.0800737	3.56E-09	0.112072	3.27E-09	0.208069
3.82E-09	-0.0479214	3.94E-09	0.144071	3.96E-09	0.048075	3.67E-09	0.560055
4.22E-09	0.144071	4.34E-09	0.144071	4.36E-09	0.0800737	4.07E-09	1.84001
4.62E-09	0.0800737	4.74E-09	-0.0799201	4.76E-09	-0.0479214	4.47E-09	2.76797
5.02E-09	-0.0159226	5.14E-09	0.17607	5.16E-09	0.0160762	4.87E-09	2.70397
5.42E-09	0.17607	5.54E-09	-0.0479214	5.56E-09	0.048075	5.27E-09	2.44798
5.82E-09	0.048075	5.94E-09	0.144071	5.96E-09	0.112072	5.67E-09	2.70397
6.22E-09	-0.0479214	6.34E-09	0.0160762	6.36E-09	0.368063	6.07E-09	3.56794
6.62E-09	0.0160762	6.74E-09	-0.0159226	6.76E-09	0.304065	6.47E-09	4.20792
7.02E-09	-0.0479214	7.14E-09	0.112072	7.16E-09	1.04004	6.87E-09	4.46391
7.42E-09	0.144071	7.54E-09	0.048075	7.56E-09	2.15999	7.27E-09	4.14392
7.82E-09	0.208069	7.94E-09	0.112072	7.96E-09	2.51198	7.67E-09	3.98392
8.22E-09	0.0160762	8.34E-09	0.112072	8.36E-09	2.28799	8.07E-09	3.79193

8.62E-09	0.208069	8.74E-09	-0.0799201	8.76E-09	2.22399	8.47E-09	3.56794
9.02E-09	-0.0799201	9.14E-09	0.144071	9.16E-09	2.60798	8.87E-09	3.34395
9.42E-09	0.144071	9.54E-09	0.144071	9.56E-09	3.40795	9.27E-09	3.05596
9.82E-09	0.0800737	9.94E-09	0.208069	9.96E-09	3.82393	9.67E-09	2.83197
1.02E-08	0.048075	1.03E-08	0.240068	1.04E-08	3.79193	1.01E-08	2.63998
1.06E-08	0.144071	1.07E-08	0.0800737	1.08E-08	3.72793	1.05E-08	2.47998
1.10E-08	-0.175916	1.11E-08	0.144071	1.12E-08	3.47194	1.09E-08	2.31999
1.14E-08	0.208069	1.15E-08	0.112072	1.16E-08	3.37595	1.13E-08	2.096
1.18E-08	0.048075	1.19E-08	0.048075	1.20E-08	3.05596	1.17E-08	2
1.22E-08	0.17607	1.23E-08	0.144071	1.24E-08	2.89597	1.21E-08	1.84001
1.26E-08	0.144071	1.27E-08	0.240068	1.28E-08	2.67197	1.25E-08	1.74401
1.30E-08	-0.0159226	1.31E-08	0.43206	1.32E-08	2.54398	1.29E-08	1.74401
1.34E-08	0.144071	1.35E-08	0.560055	1.36E-08	2.35199	1.33E-08	1.48802
1.38E-08	0.048075	1.39E-08	1.13603	1.40E-08	2.15999	1.37E-08	1.39202
1.42E-08	0.048075	1.43E-08	1.80801	1.44E-08	2.064	1.41E-08	1.26403
1.46E-08	0.17607	1.47E-08	1.968	1.48E-08	1.936	1.45E-08	1.16803
1.50E-08	0.112072	1.51E-08	1.968	1.52E-08	1.80801	1.49E-08	1.16803
1.54E-08	0.144071	1.55E-08	1.968	1.56E-08	1.68001	1.53E-08	1.00804
1.58E-08	0.048075	1.59E-08	2.25599	1.60E-08	1.48802	1.57E-08	1.00804
1.62E-08	0.112072	1.63E-08	2.79997	1.64E-08	1.45602	1.61E-08	0.944041
1.66E-08	0.208069	1.67E-08	2.92796	1.68E-08	1.26403	1.65E-08	0.848044
1.70E-08	0.208069	1.71E-08	2.95996	1.72E-08	1.16803	1.69E-08	0.880043
1.74E-08	0.304065	1.75E-08	2.89597	1.76E-08	1.16803	1.73E-08	0.752048
1.78E-08	0.43206	1.79E-08	2.70397	1.80E-08	1.00804	1.77E-08	0.720049
1.82E-08	0.912042	1.83E-08	2.51198	1.84E-08	0.912042	1.81E-08	0.592054
1.86E-08	1.32803	1.87E-08	2.31999	1.88E-08	1.00804	1.85E-08	0.592054
1.90E-08	1.58402	1.91E-08	2.22399	1.92E-08	0.816045	1.89E-08	0.592054
1.94E-08	1.39202	1.95E-08	2.064	1.96E-08	0.68805	1.93E-08	0.464059
1.98E-08	1.45602	1.99E-08	2	2.00E-08	0.656052	1.97E-08	0.528057
2.02E-08	1.74401	2.03E-08	1.904	2.04E-08	0.68805	2.01E-08	0.528057
2.06E-08	2	2.07E-08	1.77601	2.08E-08	0.656052	2.05E-08	0.400061
2.10E-08	2.19199	2.11E-08	1.68001	2.12E-08	0.560055	2.09E-08	0.400061
2.14E-08	2.22399	2.15E-08	1.55202	2.16E-08	0.560055	2.13E-08	0.400061
2.18E-08	2.19199	2.19E-08	1.42402	2.20E-08	0.464059	2.17E-08	0.400061
2.22E-08	2.032	2.23E-08	1.36002	2.24E-08	0.496058	2.21E-08	0.368063
2.26E-08	1.904	2.27E-08	1.23203	2.28E-08	0.560055	2.25E-08	0.368063
2.30E-08	1.904	2.31E-08	1.16803	2.32E-08	0.464059	2.29E-08	0.272066
2.34E-08	1.71201	2.35E-08	1.07204	2.36E-08	0.464059	2.33E-08	0.304065
2.38E-08	1.68001	2.39E-08	1.00804	2.40E-08	0.336064	2.37E-08	0.304065
2.42E-08	1.52002	2.43E-08	0.912042	2.44E-08	0.400061	2.41E-08	0.272066
2.46E-08	1.48802	2.47E-08	0.912042	2.48E-08	0.368063	2.45E-08	0.304065
2.50E-08	1.32803	2.51E-08	0.880043	2.52E-08	0.400061	2.49E-08	0.272066
2.54E-08	1.32803	2.55E-08	0.752048	2.56E-08	0.336064	2.53E-08	0.208069

2.58E-08	1.23203	2.59E-08	0.752048	2.60E-08	0.304065	2.57E-08	0.144071
2.62E-08	1.13603	2.63E-08	0.656052	2.64E-08	0.272066	2.61E-08	0.144071
2.66E-08	1.10403	2.67E-08	0.624053	2.68E-08	0.336064	2.65E-08	0.144071
2.70E-08	1.04004	2.71E-08	0.592054	2.72E-08	0.272066	2.69E-08	0.208069
2.74E-08	0.880043	2.75E-08	0.560055	2.76E-08	0.272066	2.73E-08	0.144071
2.78E-08	0.944041	2.79E-08	0.464059	2.80E-08	0.240068	2.77E-08	0.17607
2.82E-08	0.880043	2.83E-08	0.464059	2.84E-08	0.17607	2.81E-08	0.208069
2.86E-08	0.752048	2.87E-08	0.464059	2.88E-08	0.17607	2.85E-08	0.144071
2.90E-08	0.720049	2.91E-08	0.43206	2.92E-08	0.144071	2.89E-08	0.17607
2.94E-08	0.720049	2.95E-08	0.400061	2.96E-08	0.144071	2.93E-08	0.17607
2.98E-08	0.656052	2.99E-08	0.400061	3.00E-08	0.208069	2.97E-08	0.144071
3.02E-08	0.560055	3.03E-08	0.368063	3.04E-08	0.17607	3.01E-08	0.0800737
3.06E-08	0.528057	3.07E-08	0.368063	3.08E-08	0.17607	3.05E-08	0.144071
3.10E-08	0.528057	3.11E-08	0.368063	3.12E-08	0.17607	3.09E-08	0.112072
3.14E-08	0.496058	3.15E-08	0.336064	3.16E-08	0.144071	3.13E-08	0.0800737
3.18E-08	0.400061	3.19E-08	0.336064	3.20E-08	0.144071	3.17E-08	0.048075
3.22E-08	0.400061	3.23E-08	0.272066	3.24E-08	0.144071	3.21E-08	0.112072
3.26E-08	0.336064	3.27E-08	0.208069	3.28E-08	0.17607	3.25E-08	0.048075
3.30E-08	0.336064	3.31E-08	0.272066	3.32E-08	0.112072	3.29E-08	0.048075
3.34E-08	0.400061	3.35E-08	0.304065	3.36E-08	0.0800737	3.33E-08	0.0800737
3.38E-08	0.272066	3.39E-08	0.208069	3.40E-08	0.144071	3.37E-08	0.0160762
3.42E-08	0.368063	3.43E-08	0.240068	3.44E-08	0.048075	3.41E-08	0.0800737
3.46E-08	0.336064	3.47E-08	0.17607	3.48E-08	0.112072	3.45E-08	0.112072
3.50E-08	0.304065	3.51E-08	0.17607	3.52E-08	0.144071	3.49E-08	0.0160762
3.54E-08	0.304065	3.55E-08	0.17607	3.56E-08	0.112072	3.53E-08	0.144071
3.58E-08	0.304065	3.59E-08	0.112072	3.60E-08	0.0800737	3.57E-08	0.112072
3.62E-08	0.240068	3.63E-08	0.112072	3.64E-08	0.112072	3.61E-08	0.112072
3.66E-08	0.304065	3.67E-08	0.112072	3.68E-08	0.112072	3.65E-08	0.0800737
3.70E-08	0.240068	3.71E-08	0.240068	3.72E-08	0.0800737	3.69E-08	0.17607
3.74E-08	0.240068	3.75E-08	0.112072	3.76E-08	0.112072	3.73E-08	0.112072
3.78E-08	0.240068	3.79E-08	0.0800737	3.80E-08	0.144071	3.77E-08	0.0800737
3.82E-08	0.240068	3.83E-08	0.0800737	3.84E-08	0.0800737	3.81E-08	0.112072
3.86E-08	0.208069	3.87E-08	0.17607	3.88E-08	0.144071	3.85E-08	0.048075
3.90E-08	0.208069	3.91E-08	0.144071	3.92E-08	0.112072	3.89E-08	0.112072
3.94E-08	0.144071	3.95E-08	0.112072	3.96E-08	0.0800737	3.93E-08	0.112072
3.98E-08	0.144071	3.99E-08	0.112072	4.00E-08	0.048075	3.97E-08	0.048075
4.02E-08	0.048075	4.03E-08	0.112072	4.04E-08	0.0800737	4.01E-08	0.048075
4.06E-08	0.048075	4.07E-08	0.0800737	4.08E-08	0.048075	4.05E-08	0.048075
4.10E-08	0.112072	4.11E-08	0.144071	4.12E-08	0.112072	4.09E-08	0.048075
4.14E-08	0.112072	4.15E-08	0.112072	4.16E-08	0.048075	4.13E-08	0.0800737
4.18E-08	0.144071	4.19E-08	0.0800737	4.20E-08	0.0160762	4.17E-08	0.048075
4.22E-08	0.112072	4.23E-08	0.144071	4.24E-08	0.048075	4.21E-08	0.0160762
4.26E-08	0.144071	4.27E-08	0.0800737	4.28E-08	0.0160762	4.25E-08	0.112072

4.30E-08	0.0800737	4.31E-08	0.112072	4.32E-08	0.0800737	4.29E-08	0.048075
4.34E-08	0.0800737	4.35E-08	0.112072	4.36E-08	0.0800737	4.33E-08	0.0160762
4.38E-08	0.17607	4.39E-08	0.048075	4.40E-08	0.0160762	4.37E-08	0.048075
4.42E-08	0.0800737	4.43E-08	0.112072	4.44E-08	0.0160762	4.41E-08	0.048075
4.46E-08	0.0800737	4.47E-08	0.0800737	4.48E-08	-0.0159226	4.45E-08	-0.0159226
4.50E-08	0.0800737	4.51E-08	0.144071	4.52E-08	0.0160762	4.49E-08	0.048075
4.54E-08	0.144071	4.55E-08	0.0800737	4.56E-08	0.048075	4.53E-08	-0.0159226
4.58E-08	0.0800737	4.59E-08	0.048075	4.60E-08	0.0800737	4.57E-08	0.0160762
4.62E-08	0.0800737	4.63E-08	0.048075	4.64E-08	-0.0159226	4.61E-08	0.0160762
4.66E-08	0.0800737	4.67E-08	0.0800737	4.68E-08	0.0160762	4.65E-08	0.0800737
4.70E-08	0.0800737	4.71E-08	0.0160762	4.72E-08	0.048075	4.69E-08	-0.0159226
4.74E-08	0.0160762	4.75E-08	0.0800737	4.76E-08	-0.0479214	4.73E-08	0.0160762
4.78E-08	0.112072	4.79E-08	0.0800737	4.80E-08	0.0160762	4.77E-08	0.048075
4.82E-08	0.048075	4.83E-08	-0.0159226	4.84E-08	0.0160762	4.81E-08	0.0160762
4.86E-08	0.112072	4.87E-08	0.048075	4.88E-08	-0.0159226	4.85E-08	0.048075
4.90E-08	0.112072	4.91E-08	0.0800737	4.92E-08	0.0160762	4.89E-08	0.112072
4.94E-08	0.048075	4.95E-08	0.048075	4.96E-08	0.048075	4.93E-08	0.0160762
4.98E-08	0.048075	4.99E-08	0.0800737	5.00E-08	-0.0159226	4.97E-08	0.0160762
5.02E-08	0.144071	5.03E-08	-0.0479214	5.04E-08	0.048075	5.01E-08	-0.0159226
5.06E-08	0.0800737	5.07E-08	-0.0479214	5.08E-08	0.048075	5.05E-08	0.048075
5.10E-08	0.048075	5.11E-08	0.0800737	5.12E-08	0.048075	5.09E-08	0.048075
5.14E-08	0.112072	5.15E-08	-0.0159226	5.16E-08	0.0160762	5.13E-08	0.048075
5.18E-08	0.0800737	5.19E-08	-0.0159226	5.20E-08	0.0160762	5.17E-08	0.0160762
5.22E-08	0.048075	5.23E-08	0.0800737	5.24E-08	-0.0159226	5.21E-08	0.112072
5.26E-08	0.0800737	5.27E-08	0.0160762	5.28E-08	0.0800737	5.25E-08	0.0160762
5.30E-08	-0.0159226	5.31E-08	0.0160762	5.32E-08	0.0160762	5.29E-08	-0.0159226
5.34E-08	0.0800737	5.35E-08	0.048075	5.36E-08	0.0160762	5.33E-08	0.0160762
5.38E-08	0.048075	5.39E-08	0.0800737	5.40E-08	0.048075	5.37E-08	0.048075
5.42E-08	0.0160762	5.43E-08	-0.0159226	5.44E-08	0.0160762	5.41E-08	-0.0159226
5.46E-08	0.0160762	5.47E-08	0.0160762	5.48E-08	0.048075	5.45E-08	0.0160762
5.50E-08	0.0800737	5.51E-08	0.0160762	5.52E-08	0.048075	5.49E-08	0.048075
5.54E-08	-0.0159226	5.55E-08	0.0160762	5.56E-08	0.0800737	5.53E-08	0.0160762
5.58E-08	0.048075	5.59E-08	0.0160762	5.60E-08	0.0160762	5.57E-08	0.048075
5.62E-08	0.048075	5.63E-08	0.048075	5.64E-08	0.048075	5.61E-08	0.048075
5.66E-08	0.048075	5.67E-08	-0.0159226	5.68E-08	0.048075	5.65E-08	0.0160762
5.70E-08	0.048075	5.71E-08	0.0160762	5.72E-08	0.0160762	5.69E-08	0.048075
5.74E-08	0.0800737	5.75E-08	0.0160762	5.76E-08	-0.0159226	5.73E-08	0.048075
5.78E-08	0.0800737	5.79E-08	-0.0159226	5.80E-08	-0.0159226	5.77E-08	-0.0159226
5.82E-08	0.048075	5.83E-08	-0.0159226	5.84E-08	-0.0159226	5.81E-08	0.0160762
5.86E-08	0.0160762	5.87E-08	0.112072	5.88E-08	0.048075	5.85E-08	0.0800737
5.90E-08	0.0160762	5.91E-08	0.112072	5.92E-08	-0.0159226	5.89E-08	-0.0159226
5.94E-08	0.048075	5.95E-08	0.048075	5.96E-08	-0.0479214	5.93E-08	0.0800737
5.98E-08	-0.0159226	5.99E-08	0.0160762	6.00E-08	0.048075	5.97E-08	0.0160762

6.02E-08	0.0160762	6.03E-08	-0.0159226	6.04E-08	0.0160762	6.01E-08	0.0160762
6.06E-08	0.048075	6.07E-08	0.0160762	6.08E-08	-0.0159226	6.05E-08	0.0160762
6.10E-08	0.0800737	6.11E-08	0.112072	6.12E-08	0.0800737	6.09E-08	0.048075
6.14E-08	0.0160762	6.15E-08	-0.0159226	6.16E-08	0.0160762	6.13E-08	-0.0159226
6.18E-08	0.0160762	6.19E-08	-0.0159226	6.20E-08	0.0160762	6.17E-08	0.048075
6.22E-08	-0.0159226	6.23E-08	-0.0479214	6.24E-08	0.0160762	6.21E-08	-0.0159226
6.26E-08	-0.0159226	6.27E-08	-0.0159226	6.28E-08	-0.0159226	6.25E-08	-0.0159226
6.30E-08	0.048075	6.31E-08	-0.0159226	6.32E-08	-0.0479214	6.29E-08	-0.0159226
6.34E-08	0.048075	6.35E-08	-0.0159226	6.36E-08	-0.0159226	6.33E-08	0.0160762
6.38E-08	0.0160762	6.39E-08	0.048075	6.40E-08	-0.0159226	6.37E-08	0.048075
6.42E-08	-0.0479214	6.43E-08	0.0160762	6.44E-08	-0.0159226	6.41E-08	0.048075
6.46E-08	0.0160762	6.47E-08	-0.0159226	6.48E-08	-0.0479214	6.45E-08	0.048075
6.50E-08	-0.0159226	6.51E-08	0.0160762	6.52E-08	-0.0479214	6.49E-08	-0.0159226
6.54E-08	0.0800737	6.55E-08	-0.0159226	6.56E-08	-0.0159226	6.53E-08	0.0160762
6.58E-08	-0.0159226	6.59E-08	-0.0159226	6.60E-08	-0.111919	6.57E-08	0.0160762
6.62E-08	-0.0479214	6.63E-08	0.0160762	6.64E-08	-0.0479214	6.61E-08	-0.0159226
6.66E-08	0.0160762	6.67E-08	0.0160762	6.68E-08	-0.0159226	6.65E-08	0.048075
6.70E-08	0.0160762	6.71E-08	0.0160762	6.72E-08	-0.0159226	6.69E-08	0.0160762
6.74E-08	-0.0479214	6.75E-08	0.0160762	6.76E-08	-0.0159226	6.73E-08	0.048075
6.78E-08	0.0160762	6.79E-08	0.0160762	6.80E-08	0.0160762	6.77E-08	0.048075
6.82E-08	-0.0159226	6.83E-08	0.0800737	6.84E-08	-0.0159226	6.81E-08	0.0800737
6.86E-08	-0.0159226	6.87E-08	-0.0159226	6.88E-08	-0.0159226	6.85E-08	-0.0159226
6.90E-08	-0.0159226	6.91E-08	0.0160762	6.92E-08	-0.0159226	6.89E-08	0.048075
6.94E-08	0.048075	6.95E-08	0.0160762	6.96E-08	-0.0159226	6.93E-08	-0.0159226
6.98E-08	-0.0159226	6.99E-08	-0.0159226	7.00E-08	0.048075	6.97E-08	-0.0159226
7.02E-08	0.048075	7.03E-08	0.048075	7.04E-08	-0.0159226	7.01E-08	-0.0159226
7.06E-08	0.048075	7.07E-08	0.048075	7.08E-08	0.048075	7.05E-08	0.048075
7.10E-08	-0.0159226	7.11E-08	0.0160762	7.12E-08	0.0160762	7.09E-08	-0.0159226
7.14E-08	0.0160762	7.15E-08	0.0160762	7.16E-08	0.0160762	7.13E-08	0.0160762
7.18E-08	0.0160762	7.19E-08	-0.0159226	7.20E-08	-0.0159226	7.17E-08	0.0160762
7.22E-08	0.0800737	7.23E-08	-0.0479214	7.24E-08	0.048075	7.21E-08	-0.0159226
7.26E-08	0.048075	7.27E-08	0.048075	7.28E-08	0.0160762	7.25E-08	0.0160762
7.30E-08	0.0160762	7.31E-08	0.0160762	7.32E-08	-0.0159226	7.29E-08	0.048075
7.34E-08	0.0160762	7.35E-08	0.048075	7.36E-08	0.0160762	7.33E-08	-0.0159226
7.38E-08	0.0160762	7.39E-08	0.0160762	7.40E-08	-0.0479214	7.37E-08	-0.0159226
7.42E-08	0.048075	7.43E-08	0.0160762	7.44E-08	-0.0159226	7.41E-08	-0.0159226
7.46E-08	0.0160762	7.47E-08	-0.0159226	7.48E-08	0.0160762	7.45E-08	-0.0159226
7.50E-08	-0.0159226	7.51E-08	-0.0159226	7.52E-08	-0.0159226	7.49E-08	0.0800737
7.54E-08	-0.0159226	7.55E-08	-0.0159226	7.56E-08	-0.0159226	7.53E-08	-0.0799201
7.58E-08	-0.0479214	7.59E-08	-0.0479214	7.60E-08	0.048075	7.57E-08	-0.0159226
7.62E-08	-0.0159226	7.63E-08	0.0160762	7.64E-08	0.0160762	7.61E-08	0.0160762
7.66E-08	0.0160762	7.67E-08	-0.0159226	7.68E-08	-0.0159226	7.65E-08	0.0160762
7.70E-08	0.0160762	7.71E-08	0.0160762	7.72E-08	-0.0159226	7.69E-08	0.0160762

7.74E-08	-0.0159226	7.75E-08	0.0160762	7.76E-08	-0.0799201	7.73E-08	-0.0159226
7.78E-08	0.0160762	7.79E-08	-0.0159226	7.80E-08	0.0160762	7.77E-08	0.0160762
7.82E-08	-0.0159226	7.83E-08	-0.0159226	7.84E-08	-0.0479214	7.81E-08	0.048075
7.86E-08	0.0160762	7.87E-08	0.048075	7.88E-08	-0.0159226	7.85E-08	-0.0159226
7.90E-08	-0.0479214	7.91E-08	-0.0159226	7.92E-08	0.0160762	7.89E-08	0.0160762
7.94E-08	-0.0159226	7.95E-08	0.0160762	7.96E-08	0.048075	7.93E-08	-0.0479214
7.98E-08	0.0160762	7.99E-08	0.0160762	8.00E-08	-0.0159226	7.97E-08	-0.0479214
8.02E-08	-0.0159226	8.03E-08	0.0160762	8.04E-08	-0.0159226	8.01E-08	0.0160762
8.06E-08	-0.0159226	8.07E-08	-0.0159226	8.08E-08	-0.0159226	8.05E-08	-0.0159226
8.10E-08	-0.0479214	8.11E-08	0.0160762	8.12E-08	0.0800737	8.09E-08	0.048075
8.14E-08	0.0800737	8.15E-08	0.0160762	8.16E-08	0.0160762	8.13E-08	0.0160762
8.18E-08	0.0160762	8.19E-08	0.0160762	8.20E-08	-0.0479214	8.17E-08	-0.0159226
8.22E-08	0.048075	8.23E-08	-0.0159226	8.24E-08	-0.0479214	8.21E-08	-0.0479214
8.26E-08	-0.0159226	8.27E-08	-0.0159226	8.28E-08	-0.0799201	8.25E-08	-0.0159226
8.30E-08	-0.0159226	8.31E-08	-0.0479214	8.32E-08	-0.0159226	8.29E-08	-0.0159226
8.34E-08	0.0160762	8.35E-08	0.048075	8.36E-08	-0.0159226	8.33E-08	0.0160762
8.38E-08	0.0160762	8.39E-08	0.0160762	8.40E-08	0.0160762	8.37E-08	-0.0479214
8.42E-08	-0.0159226	8.43E-08	0.048075	8.44E-08	-0.0479214	8.41E-08	-0.0159226
8.46E-08	0.0160762	8.47E-08	-0.0159226	8.48E-08	-0.0159226	8.45E-08	0.0160762
8.50E-08	-0.0159226	8.51E-08	0.0800737	8.52E-08	0.0160762	8.49E-08	0.0800737
8.54E-08	0.0160762	8.55E-08	0.0160762	8.56E-08	-0.0159226	8.53E-08	-0.0159226
8.58E-08	-0.0159226	8.59E-08	-0.0159226	8.60E-08	0.048075	8.57E-08	0.048075
8.62E-08	-0.0159226	8.63E-08	0.0160762	8.64E-08	0.0160762	8.61E-08	-0.0159226
8.66E-08	-0.0479214	8.67E-08	-0.0159226	8.68E-08	-0.0159226	8.65E-08	-0.0159226
8.70E-08	-0.0159226	8.71E-08	0.0160762	8.72E-08	0.0160762	8.69E-08	0.0160762
8.74E-08	0.048075	8.75E-08	0.048075	8.76E-08	0.0160762	8.73E-08	-0.0159226
8.78E-08	-0.0159226	8.79E-08	-0.0159226	8.80E-08	-0.0799201	8.77E-08	-0.0159226
8.82E-08	-0.0159226	8.83E-08	0.0160762	8.84E-08	-0.0159226	8.81E-08	0.0160762
8.86E-08	-0.0159226	8.87E-08	0.0160762	8.88E-08	0.0160762	8.85E-08	-0.0159226
8.90E-08	-0.0479214	8.91E-08	-0.0159226	8.92E-08	0.0800737	8.89E-08	-0.0159226
8.94E-08	0.0160762	8.95E-08	-0.0159226	8.96E-08	-0.0159226	8.93E-08	-0.0799201
8.98E-08	0.0160762	8.99E-08	0.0160762	9.00E-08	0.048075	8.97E-08	-0.0159226
9.02E-08	-0.0159226	9.03E-08	-0.0159226	9.04E-08	0.048075	9.01E-08	-0.0159226
9.06E-08	0.0160762	9.07E-08	-0.0159226	9.08E-08	-0.0159226	9.05E-08	0.0160762
9.10E-08	0.0160762	9.11E-08	-0.0479214	9.12E-08	0.0160762	9.09E-08	0.0160762
9.14E-08	0.0160762	9.15E-08	-0.0159226	9.16E-08	-0.0159226	9.13E-08	-0.0159226
9.18E-08	-0.0159226	9.19E-08	-0.0159226	9.20E-08	-0.0479214	9.17E-08	-0.0159226
9.22E-08	-0.0159226	9.23E-08	0.0160762	9.24E-08	-0.0159226	9.21E-08	0.0160762
9.26E-08	0.0160762	9.27E-08	-0.0159226	9.28E-08	-0.0159226	9.25E-08	-0.0159226
9.30E-08	-0.0159226	9.31E-08	-0.0159226	9.32E-08	-0.0159226	9.29E-08	0.0160762
9.34E-08	-0.0159226	9.35E-08	0.048075	9.36E-08	-0.0159226	9.33E-08	-0.0479214
9.38E-08	0.0160762	9.39E-08	-0.0159226	9.40E-08	0.0160762	9.37E-08	0.0160762
9.42E-08	0.0160762	9.43E-08	-0.0159226	9.44E-08	-0.0159226	9.41E-08	-0.0159226

9.46E-08	0.048075	9.47E-08	-0.0159226	9.48E-08	0.0160762	9.45E-08	0.0160762
9.50E-08	-0.0479214	9.51E-08	-0.0159226	9.52E-08	0.0160762	9.49E-08	-0.0479214
9.54E-08	0.0160762	9.55E-08	-0.0159226	9.56E-08	-0.0479214	9.53E-08	-0.0479214
9.58E-08	-0.0159226	9.59E-08	-0.0159226	9.60E-08	-0.0159226	9.57E-08	-0.0799201
9.62E-08	0.0160762	9.63E-08	-0.0479214	9.64E-08	0.0160762	9.61E-08	-0.0159226
9.66E-08	0.0160762	9.67E-08	-0.0159226	9.68E-08	-0.0159226	9.65E-08	0.0160762
9.70E-08	0.048075	9.71E-08	0.0800737	9.72E-08	-0.0159226	9.69E-08	0.048075
9.74E-08	-0.0159226	9.75E-08	0.0160762	9.76E-08	-0.0159226	9.73E-08	0.0160762
9.78E-08	0.0800737	9.79E-08	0.0160762	9.80E-08	-0.0159226	9.77E-08	0.048075
9.82E-08	-0.0479214	9.83E-08	0.048075	9.84E-08	-0.0479214	9.81E-08	0.0160762
9.86E-08	-0.0479214	9.87E-08	-0.0159226	9.88E-08	0.0160762	9.85E-08	-0.0159226
9.90E-08	0.0160762	9.91E-08	0.0160762	9.92E-08	-0.0159226	9.89E-08	-0.0159226
9.94E-08	0.0160762	9.95E-08	-0.0799201	9.96E-08	0.0160762	9.93E-08	0.0160762
9.98E-08	-0.0159226	9.99E-08	-0.0159226	1.00E-07	-0.0479214	9.97E-08	-0.0159226
1.00E-07	0.0160762	1.00E-07	0.112072	1.00E-07	0.0160762	1.00E-07	0.048075
1.01E-07	0.0800737	1.01E-07	-0.0159226	1.01E-07	-0.0159226	1.00E-07	-0.0479214
1.01E-07	-0.0159226	1.01E-07	0.0160762	1.01E-07	0.048075	1.01E-07	-0.0159226
1.01E-07	0.0800737	1.02E-07	0.048075	1.02E-07	0.0160762	1.01E-07	0.0160762
1.02E-07	0.0800737	1.02E-07	-0.0159226	1.02E-07	0.0160762	1.02E-07	-0.0479214
1.02E-07	-0.0479214	1.02E-07	0.0160762	1.02E-07	-0.0159226	1.02E-07	-0.0799201
1.03E-07	-0.0479214	1.03E-07	0.048075	1.03E-07	-0.0159226	1.02E-07	0.0160762
1.03E-07	-0.0159226	1.03E-07	0.0160762	1.03E-07	0.0160762	1.03E-07	-0.0159226
1.03E-07	-0.0479214	1.04E-07	-0.0159226	1.04E-07	0.0160762	1.03E-07	-0.0159226
1.04E-07	0.0160762	1.04E-07	-0.0159226	1.04E-07	-0.0479214	1.04E-07	0.0160762
1.04E-07	0.048075	1.04E-07	0.0160762	1.04E-07	0.0160762	1.04E-07	0.0800737
1.05E-07	-0.0159226	1.05E-07	-0.0479214	1.05E-07	0.0160762	1.04E-07	0.0160762
1.05E-07	-0.0159226	1.05E-07	0.0160762	1.05E-07	0.0160762	1.05E-07	0.0160762
1.05E-07	0.0160762	1.06E-07	-0.0159226	1.06E-07	0.048075	1.05E-07	0.0160762
1.06E-07	-0.0479214	1.06E-07	-0.0159226	1.06E-07	-0.0479214	1.06E-07	-0.0159226
1.06E-07	0.0160762	1.06E-07	-0.0159226	1.06E-07	-0.0479214	1.06E-07	0.0160762
1.07E-07	-0.0159226	1.07E-07	-0.0159226	1.07E-07	0.0160762	1.06E-07	-0.0159226
1.07E-07	0.0160762	1.07E-07	-0.0159226	1.07E-07	0.0160762	1.07E-07	0.0160762
1.07E-07	0.048075	1.08E-07	0.048075	1.08E-07	-0.0159226	1.07E-07	0.0160762
1.08E-07	0.0160762	1.08E-07	-0.0159226	1.08E-07	0.048075	1.08E-07	-0.0479214
1.08E-07	0.0160762	1.08E-07	-0.0159226	1.08E-07	-0.0159226	1.08E-07	-0.0159226
1.09E-07	0.048075	1.09E-07	0.0160762	1.09E-07	-0.0159226	1.08E-07	0.0160762
1.09E-07	0.0160762	1.09E-07	0.048075	1.09E-07	-0.0159226	1.09E-07	-0.0159226
1.09E-07	0.048075	1.10E-07	-0.0159226	1.10E-07	-0.0159226	1.09E-07	-0.0159226
1.10E-07	0.0160762	1.10E-07	0.048075	1.10E-07	-0.0159226	1.10E-07	0.0160762
1.10E-07	-0.0159226	1.10E-07	0.0160762	1.10E-07	-0.0159226	1.10E-07	0.0160762
1.11E-07	-0.0479214	1.11E-07	-0.0159226	1.11E-07	0.0160762	1.10E-07	0.0160762
1.11E-07	0.0160762	1.11E-07	0.0160762	1.11E-07	-0.0159226	1.11E-07	0.0160762
1.11E-07	-0.0159226	1.12E-07	0.0160762	1.12E-07	0.048075	1.11E-07	0.048075

1.12E-07	-0.0479214	1.12E-07	0.048075	1.12E-07	-0.0159226	1.12E-07	-0.0159226
1.12E-07	0.0160762	1.12E-07	0.0160762	1.12E-07	0.0160762	1.12E-07	-0.0159226
1.13E-07	-0.0479214	1.13E-07	-0.0159226	1.13E-07	-0.0159226	1.12E-07	-0.0159226
1.13E-07	-0.0159226	1.13E-07	-0.0159226	1.13E-07	-0.0799201	1.13E-07	-0.0479214
1.13E-07	0.0160762	1.14E-07	0.0160762	1.14E-07	-0.0159226	1.13E-07	-0.0159226
1.14E-07	-0.0159226	1.14E-07	0.0160762	1.14E-07	-0.0159226	1.14E-07	-0.0159226
1.14E-07	-0.0159226	1.14E-07	-0.0159226	1.14E-07	-0.0799201	1.14E-07	-0.0479214
1.15E-07	-0.0159226	1.15E-07	0.048075	1.15E-07	-0.0479214	1.14E-07	0.0160762
1.15E-07	-0.0479214	1.15E-07	0.0160762	1.15E-07	0.0160762	1.15E-07	-0.0479214
1.15E-07	-0.0479214	1.16E-07	0.0160762	1.16E-07	0.048075	1.15E-07	-0.0479214
1.16E-07	0.048075	1.16E-07	0.048075	1.16E-07	0.0160762	1.16E-07	-0.0159226
1.16E-07	0.0800737	1.16E-07	0.0800737	1.16E-07	-0.0159226	1.16E-07	-0.0799201
1.17E-07	0.0160762	1.17E-07	0.0160762	1.17E-07	0.048075	1.16E-07	-0.0799201
1.17E-07	0.048075	1.17E-07	0.0800737	1.17E-07	-0.0159226	1.17E-07	-0.0159226
1.17E-07	0.0160762	1.18E-07	0.0160762	1.18E-07	0.0160762	1.17E-07	-0.0159226
1.18E-07	-0.0479214	1.18E-07	-0.0799201	1.18E-07	0.0160762	1.18E-07	-0.0479214
1.18E-07	0.0160762	1.18E-07	-0.0159226	1.18E-07	-0.0159226	1.18E-07	0.0160762
1.19E-07	-0.0159226	1.19E-07	0.048075	1.19E-07	0.048075	1.18E-07	0.0160762
1.19E-07	-0.0479214	1.19E-07	0.048075	1.19E-07	0.0800737	1.19E-07	-0.0159226
1.19E-07	-0.0159226	1.20E-07	0.0160762	1.20E-07	-0.0159226	1.19E-07	-0.0479214
1.20E-07	0.0160762	1.20E-07	0.048075	1.20E-07	-0.0159226	1.20E-07	0.0160762
1.20E-07	-0.0479214	1.20E-07	-0.0159226	1.20E-07	-0.0159226	1.20E-07	-0.0159226
1.21E-07	-0.0159226	1.21E-07	0.0160762	1.21E-07	0.0160762	1.20E-07	-0.0479214
1.21E-07	0.0160762	1.21E-07	0.0160762	1.21E-07	0.0160762	1.21E-07	-0.0159226
1.21E-07	-0.0159226	1.22E-07	-0.0159226	1.22E-07	-0.0479214	1.21E-07	-0.0479214
1.22E-07	-0.0159226	1.22E-07	0.048075	1.22E-07	-0.0159226	1.22E-07	-0.0159226
1.22E-07	-0.0159226	1.22E-07	-0.0159226	1.22E-07	-0.0159226	1.22E-07	-0.0159226
1.23E-07	-0.0159226	1.23E-07	-0.0159226	1.23E-07	0.0160762	1.22E-07	-0.0159226
1.23E-07	-0.0159226	1.23E-07	0.0160762	1.23E-07	0.0160762	1.23E-07	0.0160762
1.23E-07	-0.0479214	1.24E-07	0.0160762	1.24E-07	-0.0159226	1.23E-07	0.048075
1.24E-07	-0.0479214	1.24E-07	-0.0159226	1.24E-07	0.048075	1.24E-07	-0.0479214
1.24E-07	0.0160762	1.24E-07	-0.0159226	1.24E-07	-0.0159226	1.24E-07	-0.0159226
1.25E-07	0.0160762	1.25E-07	0.048075	1.25E-07	-0.0159226	1.24E-07	0.0160762
1.25E-07	0.0160762	1.25E-07	-0.0159226	1.25E-07	-0.0159226	1.25E-07	0.0160762
1.25E-07	0.0160762	1.26E-07	0.048075	1.26E-07	-0.0479214	1.25E-07	0.048075
1.26E-07	0.0160762	1.26E-07	-0.0159226	1.26E-07	0.0160762	1.26E-07	0.0160762
1.26E-07	0.0160762	1.26E-07	0.0160762	1.26E-07	-0.0479214	1.26E-07	-0.0159226
1.27E-07	0.0160762	1.27E-07	-0.0159226	1.27E-07	-0.0159226	1.26E-07	0.048075
1.27E-07	0.048075	1.27E-07	-0.0479214	1.27E-07	-0.0479214	1.27E-07	0.048075
1.27E-07	-0.0159226	1.28E-07	-0.0159226	1.28E-07	0.048075	1.27E-07	-0.0479214
1.28E-07	-0.0159226	1.28E-07	0.0160762	1.28E-07	0.0160762	1.28E-07	0.048075
1.28E-07	0.0160762	1.28E-07	0.048075	1.28E-07	0.048075	1.28E-07	-0.0159226
1.29E-07	0.0160762	1.29E-07	-0.0159226	1.29E-07	0.0160762	1.28E-07	-0.0479214

1.29E-07	-0.0159226	1.29E-07	0.0160762	1.29E-07	0.0160762	1.29E-07	0.0160762
1.29E-07	-0.0159226	1.30E-07	-0.0479214	1.30E-07	0.0160762	1.29E-07	0.0160762
1.30E-07	-0.0159226	1.30E-07	-0.0159226	1.30E-07	-0.0159226	1.30E-07	0.048075
1.30E-07	0.0160762	1.30E-07	-0.0159226	1.30E-07	-0.0159226	1.30E-07	-0.0159226
1.31E-07	0.0160762	1.31E-07	0.0160762	1.31E-07	0.048075	1.30E-07	-0.0159226
1.31E-07	0.0160762	1.31E-07	-0.0159226	1.31E-07	0.0160762	1.31E-07	-0.0159226
1.31E-07	0.0160762	1.32E-07	0.0800737	1.32E-07	0.0160762	1.31E-07	0.0160762
1.32E-07	-0.0159226	1.32E-07	-0.0159226	1.32E-07	-0.0159226	1.32E-07	0.0160762
1.32E-07	0.0160762	1.32E-07	-0.0159226	1.32E-07	-0.0479214	1.32E-07	0.048075
1.33E-07	0.0160762	1.33E-07	-0.0159226	1.33E-07	0.048075	1.32E-07	0.0160762
1.33E-07	-0.0159226	1.33E-07	-0.0159226	1.33E-07	0.048075	1.33E-07	0.048075
1.33E-07	-0.0159226	1.34E-07	0.0160762	1.34E-07	0.048075	1.33E-07	0.0160762
1.34E-07	-0.0479214	1.34E-07	0.048075	1.34E-07	0.0160762	1.34E-07	0.0160762
1.34E-07	0.0160762	1.34E-07	0.048075	1.34E-07	0.0160762	1.34E-07	-0.0159226
1.35E-07	-0.0479214	1.35E-07	-0.0159226	1.35E-07	-0.0159226	1.34E-07	0.0160762
1.35E-07	0.0800737	1.35E-07	0.048075	1.35E-07	-0.0159226	1.35E-07	0.0160762
1.35E-07	0.0160762	1.36E-07	-0.0159226	1.36E-07	0.0160762	1.35E-07	0.0160762
1.36E-07	0.048075	1.36E-07	-0.0159226	1.36E-07	0.048075	1.36E-07	-0.0159226
1.36E-07	0.0160762	1.36E-07	-0.0159226	1.36E-07	-0.0159226	1.36E-07	0.0160762
1.37E-07	0.0160762	1.37E-07	0.048075	1.37E-07	0.0160762	1.36E-07	-0.0159226
1.37E-07	-0.0159226	1.37E-07	-0.0479214	1.37E-07	0.0160762	1.37E-07	-0.0159226
1.37E-07	0.0160762	1.38E-07	0.0160762	1.38E-07	-0.0159226	1.37E-07	-0.0159226
1.38E-07	0.0160762	1.38E-07	0.0160762	1.38E-07	0.0160762	1.38E-07	-0.0479214
1.38E-07	0.0160762	1.38E-07	-0.0159226	1.38E-07	0.048075	1.38E-07	-0.0159226
1.39E-07	0.0160762	1.39E-07	-0.0159226	1.39E-07	-0.0159226	1.38E-07	0.0160762
1.39E-07	0.0160762	1.39E-07	0.048075	1.39E-07	-0.0159226	1.39E-07	-0.0159226
1.39E-07	-0.0159226	1.40E-07	0.0160762	1.40E-07	0.0800737	1.39E-07	-0.0159226
1.40E-07	-0.0159226	1.40E-07	0.0160762	1.40E-07	-0.0159226	1.40E-07	0.0160762
1.40E-07	-0.0159226	1.40E-07	0.0160762	1.40E-07	0.0160762	1.40E-07	0.0160762
1.41E-07	-0.0159226	1.41E-07	-0.0159226	1.41E-07	0.0160762	1.40E-07	-0.0159226
1.41E-07	0.0160762	1.41E-07	0.0160762	1.41E-07	-0.0159226	1.41E-07	0.0800737
1.41E-07	0.0800737	1.42E-07	0.048075	1.42E-07	0.0160762	1.41E-07	-0.0159226
1.42E-07	0.0160762	1.42E-07	0.0160762	1.42E-07	0.048075	1.42E-07	-0.0159226
1.42E-07	-0.0159226	1.42E-07	0.0160762	1.42E-07	-0.0159226	1.42E-07	0.048075
1.43E-07	0.0160762	1.43E-07	0.048075	1.43E-07	-0.0159226	1.42E-07	0.0160762
1.43E-07	-0.0159226	1.43E-07	0.0160762	1.43E-07	-0.0159226	1.43E-07	0.0160762
1.43E-07	-0.0159226	1.44E-07	0.0160762	1.44E-07	0.0160762	1.43E-07	-0.0159226
1.44E-07	0.0160762	1.44E-07	0.0160762	1.44E-07	-0.0159226	1.44E-07	-0.0479214
1.44E-07	0.0160762	1.44E-07	-0.0479214	1.44E-07	0.048075	1.44E-07	0.0160762
1.45E-07	0.0800737	1.45E-07	-0.0479214	1.45E-07	-0.0159226	1.44E-07	0.0160762
1.45E-07	0.048075	1.45E-07	-0.0159226	1.45E-07	0.0160762	1.45E-07	0.0160762
1.45E-07	0.0160762	1.46E-07	0.0160762	1.46E-07	0.0160762	1.45E-07	-0.0479214
1.46E-07	0.0800737	1.46E-07	-0.0159226	1.46E-07	-0.0479214	1.46E-07	0.0160762

1.46E-07	0.0160762	1.46E-07	0.0160762	1.46E-07	0.0160762	1.46E-07	-0.0479214
1.47E-07	-0.0159226	1.47E-07	-0.0159226	1.47E-07	0.0160762	1.46E-07	-0.0159226
1.47E-07	0.048075	1.47E-07	-0.0159226	1.47E-07	0.0160762	1.47E-07	-0.0159226
1.47E-07	-0.0479214	1.48E-07	0.048075	1.48E-07	0.0160762	1.47E-07	0.048075
1.48E-07	-0.0479214	1.48E-07	0.0160762	1.48E-07	0.048075	1.48E-07	-0.0159226
1.48E-07	0.0160762	1.48E-07	0.0160762	1.48E-07	-0.0159226	1.48E-07	0.0160762
1.49E-07	0.0160762	1.49E-07	-0.0159226	1.49E-07	-0.0159226	1.48E-07	-0.0159226
1.49E-07	0.0160762	1.49E-07	0.048075	1.49E-07	-0.0159226	1.49E-07	0.0160762
1.49E-07	0.0160762	1.50E-07	0.0160762	1.50E-07	0.0160762	1.49E-07	-0.0159226
1.50E-07	0.0160762	1.50E-07	0.0160762	1.50E-07	0.0160762	1.50E-07	0.0800737
1.50E-07	-0.0479214	1.50E-07	-0.0159226	1.50E-07	0.048075	1.50E-07	0.048075
1.51E-07	0.048075	1.51E-07	0.048075	1.51E-07	0.0160762	1.50E-07	0.0160762
1.51E-07	0.112072	1.51E-07	0.0160762	1.51E-07	0.048075	1.51E-07	0.048075
1.51E-07	0.0160762	1.52E-07	-0.0159226	1.52E-07	0.0160762	1.51E-07	-0.0159226
1.52E-07	-0.0159226	1.52E-07	0.0160762	1.52E-07	-0.0479214	1.52E-07	0.048075
1.52E-07	-0.0159226	1.52E-07	0.0160762	1.52E-07	0.0160762	1.52E-07	0.0800737
1.53E-07	-0.0159226	1.53E-07	0.0160762	1.53E-07	0.048075	1.52E-07	0.0160762
1.53E-07	0.0160762	1.53E-07	0.048075	1.53E-07	0.0160762	1.53E-07	0.0160762
1.53E-07	0.048075	1.54E-07	-0.0159226	1.54E-07	0.0160762	1.53E-07	-0.0159226
1.54E-07	-0.0479214	1.54E-07	-0.0479214	1.54E-07	0.0160762	1.54E-07	-0.0159226
1.54E-07	-0.0159226	1.54E-07	0.048075	1.54E-07	0.0160762	1.54E-07	0.0160762
1.55E-07	0.0160762	1.55E-07	0.0160762	1.55E-07	0.048075	1.54E-07	0.0160762
1.55E-07	-0.0159226	1.55E-07	-0.0159226	1.55E-07	-0.0159226	1.55E-07	0.0160762
1.55E-07	-0.0159226	1.56E-07	0.0160762	1.56E-07	-0.0159226	1.55E-07	0.0160762
1.56E-07	0.048075	1.56E-07	0.0160762	1.56E-07	0.0160762	1.56E-07	0.048075
1.56E-07	-0.0159226	1.56E-07	-0.0479214	1.56E-07	-0.0159226	1.56E-07	-0.0159226
1.57E-07	0.0160762	1.57E-07	-0.0159226	1.57E-07	-0.0159226	1.56E-07	-0.0159226
1.57E-07	-0.0159226	1.57E-07	0.0160762	1.57E-07	0.0160762	1.57E-07	0.048075
1.57E-07	0.0160762	1.58E-07	-0.0159226	1.58E-07	0.0160762	1.57E-07	0.0160762
1.58E-07	0.0160762	1.58E-07	0.0160762	1.58E-07	0.0160762	1.58E-07	0.0160762
1.58E-07	0.0800737	1.58E-07	0.048075	1.58E-07	-0.0159226	1.58E-07	0.048075
1.59E-07	-0.0159226	1.59E-07	0.0160762	1.59E-07	0.0800737	1.58E-07	-0.0159226
1.59E-07	0.0800737	1.59E-07	0.048075	1.59E-07	-0.0479214	1.59E-07	0.048075
1.59E-07	-0.0159226	1.60E-07	-0.0159226	1.60E-07	0.0160762	1.59E-07	0.048075
1.60E-07	0.0160762	1.60E-07	-0.0479214	1.60E-07	0.0160762	1.60E-07	-0.0159226
1.60E-07	0.0160762	1.60E-07	0.048075	1.60E-07	-0.0479214	1.60E-07	-0.0159226
